# AGE DIFFERENCES IN WOMEN’S EMPATHIC ACCURACY WITHIN LONG-TERM MARRIAGES

**DOI:** 10.1093/geroni/igae098.1483

**Published:** 2024-12-31

**Authors:** Casey Brown, Jenna Wells, Claudia Haase, Robert Levenson

**Affiliations:** Georgetown University, Washington, District of Columbia, United States; Cornell University, Ithaca, New York, United States; Northwestern University, Evanston, Illinois, United States; University of California Berkeley, Berkeley, California, United States

## Abstract

Understanding other people’s emotions accurately (i.e., empathic accuracy) is thought to be critical for building and maintaining social connections. Past research suggests empathic skills change with age, but few studies examine age differences in empathic accuracy within the context of close relationships. We examined whether empathic accuracy is higher among middle-aged couples (ages 40-50) compared to older couples (ages 60-70) using a sample of 154 heterosexual long-term marriages. Husbands and wives visited the laboratory and engaged in a 15-minute conversation on a topic of disagreement in their marriage. Conversations were video-recorded. Husbands and wives watched a video playback of their conversation twice, each time continuously rating either their own or their spouse’s emotional valence during the conversation using a rating dial that ranged from “very negative” to “very positive”. These continuous valence ratings were used to compute each spouse’s empathic accuracy as the strength of the correlation between their ratings of their spouse’s emotions and their spouse’s own self- ratings. Results revealed that age was not associated with husbands’ empathic accuracy. In contrast, older wives had greater empathic accuracy compared to middle-aged wives (Mdiff =.19, p =.028). However, older adults had been married longer, and length of marriage also predicted women’s empathic accuracy. Findings suggest that older women are better able to track the changing valence of their husbands’ emotions, perhaps because they have more practice.

